# Ecosystem state change in the Arabian Sea fuelled by the recent loss of snow over the Himalayan-Tibetan Plateau region

**DOI:** 10.1038/s41598-020-64360-2

**Published:** 2020-05-04

**Authors:** Joaquim I. Goes, Hongzhen Tian, Helga do Rosario Gomes, O. Roger Anderson, Khalid Al-Hashmi, Sergio deRada, Hao Luo, Lubna Al-Kharusi, Adnan Al-Azri, Douglas G. Martinson

**Affiliations:** 1 Lamont Doherty Earth Observatory at Columbia University, Palisades, New York 10964 USA; 2 College of Economics and Management, Tiangong University, 399 Binshui West Road, Tianjin, 300387 P.R. China; 3 Department of Marine Science and Fisheries, Sultan Qaboos University, Al-Khoud, Muscat, 123, Sultanate of Oman; 4 Naval Research Laboratory, Stennis Space Center, Mississippi, MS 39529 USA; 5 State Key Laboratory of Marine Environmental Science and College of Ocean and Earth Sciences, Xiamen University, Xiamen, 361000 China; 6 Ministry of Fisheries and Agricultural Wealth, Muscat, 100 Sultanate of Oman; 7 Ministry of Foreign Affairs, Muscat, 100 Sultanate of Oman

**Keywords:** Climate sciences, Marine biology

## Abstract

The recent trend of global warming has exerted a disproportionately strong influence on the Eurasian land surface, causing a steady decline in snow cover extent over the Himalayan-Tibetan Plateau region. Here we show that this loss of snow is undermining winter convective mixing and causing stratification of the upper layer of the Arabian Sea at a much faster rate than predicted by global climate models. Over the past four decades, the Arabian Sea has also experienced a profound loss of inorganic nitrate. In all probability, this is due to increased denitrification caused by the expansion of the permanent oxygen minimum zone and consequent changes in nutrient stoichiometries. These exceptional changes appear to be creating a niche particularly favorable to the mixotroph, *Noctiluca scintillans* which has recently replaced diatoms as the dominant winter, bloom forming organism. Although *Noctiluca* blooms are non-toxic, they can cause fish mortality by exacerbating oxygen deficiency and ammonification of seawater. As a consequence, their continued range expansion represents a significant and growing threat for regional fisheries and the welfare of coastal populations dependent on the Arabian Sea for sustenance.

## Introduction

The Arabian Sea (AS) is a unique, low-latitude oceanic ecosystem because it is influenced by monsoonal winds that reverse their direction seasonally^1^. These reversing winds cause dynamic shifts in surface currents and alterations in the pycnocline, which help fertilize its normally nutrient-depleted surface layers. However, the mechanisms that drive water-column mixing, the upward transport of nutrients and the consequent upsurge of phytoplankton biomass during the summer (Jun.-Sep.) and the winter (Nov.-Feb.) monsoons are different^2–6^. During the summer monsoon, nutrient enrichment is via coastal upwelling, which begins when the adjacent land mass becomes warmer relative to the AS, and low pressure develops over the Arabian Peninsula^3,7,8^. During this time of the year, strong topographically-steered southwesterly winds blowing over the AS form a low-level atmospheric jet called the Findlater Jet^9^. This jet induces a northeastwardly flow of the surface currents, leading to strong upwelling of deep, nutrient-rich waters, causing large phytoplankton blooms along the coasts of Somalia, Yemen, and Oman^3,5,6,10,11^. In contrast, during winter, when the Eurasian continent cools, nutrient enrichment is via deep penetrative convective mixing as a result of excessive heat loss from the AS surface layer caused by cold and dry northeasterly winds blowing from the snow-covered Himalayan-Tibetan Plateau (HTP)^4,5^. The erosion of the thermocline, and entrainment of deep (100–150 m) nutrient-rich waters into the surface layer of the AS, begins as early as Dec., and has a major influence on winter phytoplankton blooms observable as far south as 14°N^4,11,12^.

The impacts of anthropogenic climate change on the monsoonal system have elicited a large number of studies in recent years^8,13,14^. Most of these have focused on the summer monsoon, because of its implications for rainfall patterns and water supply in countries downstream of the summer monsoon^13,15–17^; and especially because of its consequences for coastal upwelling, biological productivity and fisheries in countries bordering the AS^3^. In contrast, the response of the boreal winter component of the monsoon cycle to global warming has received far less attention, despite knowledge that it has a significant impact on annual precipitation patterns over the HTP region and over southeast India and Sri Lanka^18^, and on winter convective mixing, responsible for sustaining the large phytoplankton blooms of winter, which contribute significantly to enhancing the rich fisheries potential of the AS^4,5,12,19^.

A recent study^20^, based on results from Coupled Model Intercomparison Project Phase 5 (CMIP5), predicts that the AS will experience significant weakening of winter monsoon winds by the turn of the 21st century due to enhanced warming of the dry Arabian Peninsula relative to the southern Indian Ocean. Weaker winds would result in a substantial reduction in oceanic heat loss to the atmosphere and robust weakening of convective mixing, which the authors^20^ postulate would lead to a reduction in the productivity and size of winter phytoplankton blooms in the AS.

Here we present data on more contemporaneous changes in winter convective mixing using mixed layer depth (MLD) outputs from the Global Ocean Data Assimilation System (GODAS)^21^, a real time analysis and reanalysis system used for retrospective analysis, and for monitoring of present-day oceanic conditions. Using contemporary Chlorophyll *a* (Chl *a*, a proxy for phytoplankton biomass) data obtained from satellite and field studies, we attempt to explain why the response of the AS ecosystem under a scenario of weaker winter convective mixing and enhanced stratification, differs substantially from what has been projected in previous studies^20,22^.

## Results and Discussion

**Figure 1:**
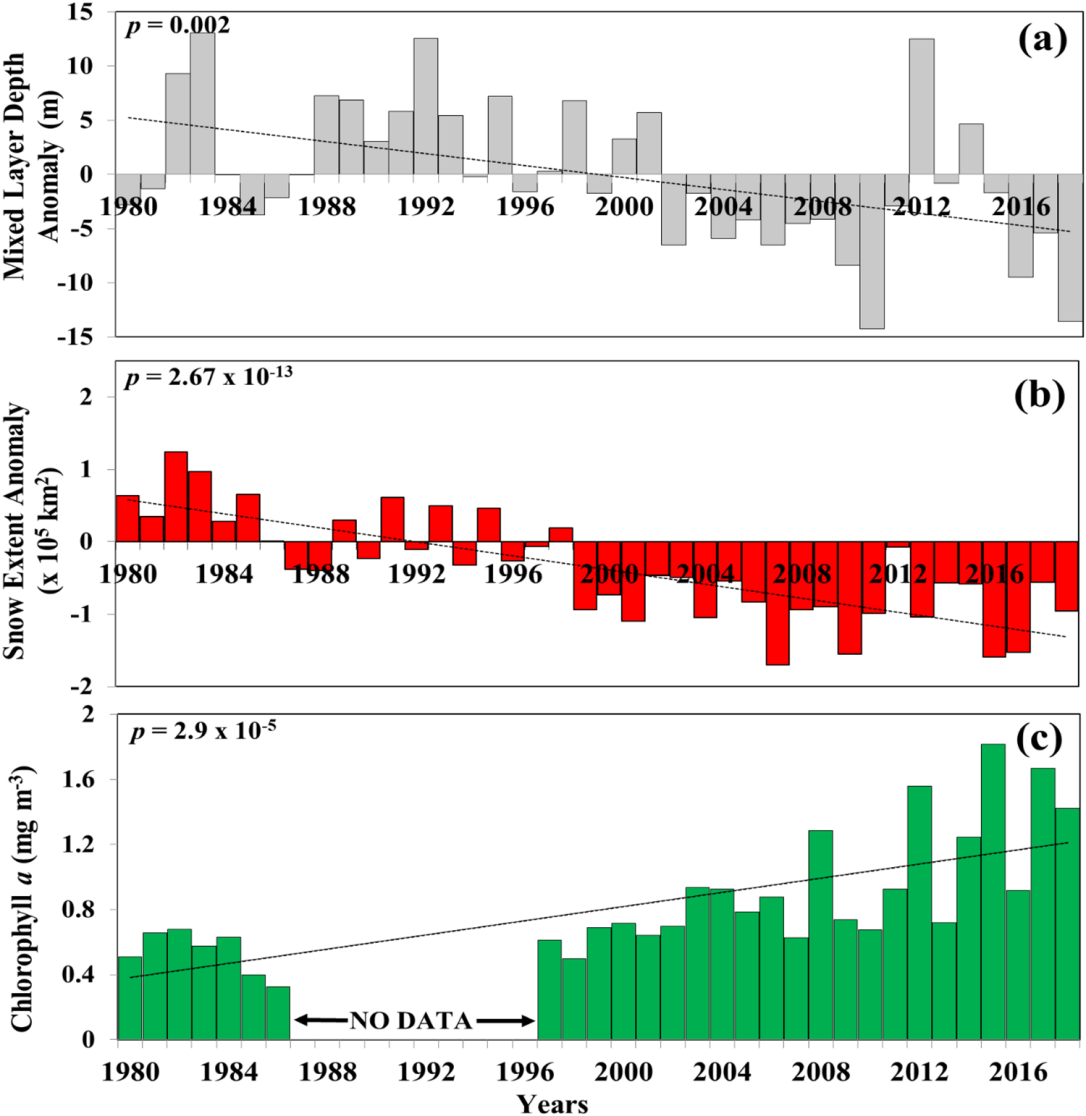
Time series of winter-time, AS area-averaged anomalies (departures from means) of: (**a**) mixed layer depth, (**b**) HTP snow cover extent, (**c**) Chl *a* concentrations (not anomaly). Trends (dashed lines) were obtained using linear least square regression fits to the data. Also shown in Figs are *p* values associated with individual trends *(Additional details in methods section)*. All the data processing was conducted using the cloud computing technology in the Google Earth Engine (GEE) platform (https://earthengine.google.org/)^59^.

Consistent with CMIP5 projections shown earlier^20^, MLD trends from GODAS also show a weakening of winter convective mixing (Fig. 1a), but notably at a rate (0.28 m yr^−1^) much faster than the 0.15 m yr^−1^ rate predicted in the CMIP5-based study^20^. Despite certain limitations^21,23,24^, GODAS provides more realistic and robust outputs of MLDs than CMIP5, because it is a real-time ocean analysis and reanalysis system, in which model outputs are constrained by *in-situ* observations, unlike the unconstrained ensemble climate projections of CMIP5. Outputs from GODAS show that since 1980, winter-time average AS basin-wide MLDs have decreased by>11 m, a change that has been accompanied by a warming of winter monsoon winds (~0.012 °C yr^−1^), a decline in the strength of these winds (~0.006 m s^−1^ yr^−1^) and an increase in their relative humidity (0.1% yr^−1^) (Fig. S1a–c). Collectively, these changes have resulted in an increase in net-heat flux from the atmosphere into AS surface waters (Fig. S1d) that indicates an increase in the upper AS ocean heat content (OHC) since 2000^25^. Convective mixing is driven primarily by buoyancy destabilizing forces that include cooling and/or evaporation and that cause surface ocean waters to become colder, saltier and denser than the underlying waters. The process is greatly enhanced when the overlying winds are colder, drier and stronger, but conversely, weakened when they become warmer and more humid as witnessed recently in the AS (Fig. S1a–c).

The temperature of winter winds and their dryness is governed in large part by the extent of winter snow cover over the HTP^10–12^, which also impacts land-ocean temperature and pressure gradients that modulate the strength of the winter monsoonal winds^15,16,18–20^. Since 1998, the HTP has experienced a persistent year-on-year decline (~5 ×10^3^ yr^−1^) in snow cover extent^26^ (Fig. 1b), attributable to the warming of the Eurasian continent (Fig. S2)^3^, a trend that has possibly been exacerbated by the deposition of soot and dust on the HTP snow surface^27^. Regression analysis of HTP snow cover extent versus average mixed layer depths indicates that the loss of snow accounts for around 51% of the recent shallowing trends in the mixed layer of the AS (Fig. S3, r^2^ = 0.51, *p* < 0.0001), through generation of warmer, weaker and more humid offshore-blowing winds that result in an increase in net heat flux (Fig. S1a–d). Cumulatively the major consequence of these changes is the undermining of convective mixing responsible for nutrient entrainment into the euphotic zone and the fertilization of large winter phytoplankton blooms^4^.

**Figure 2:**
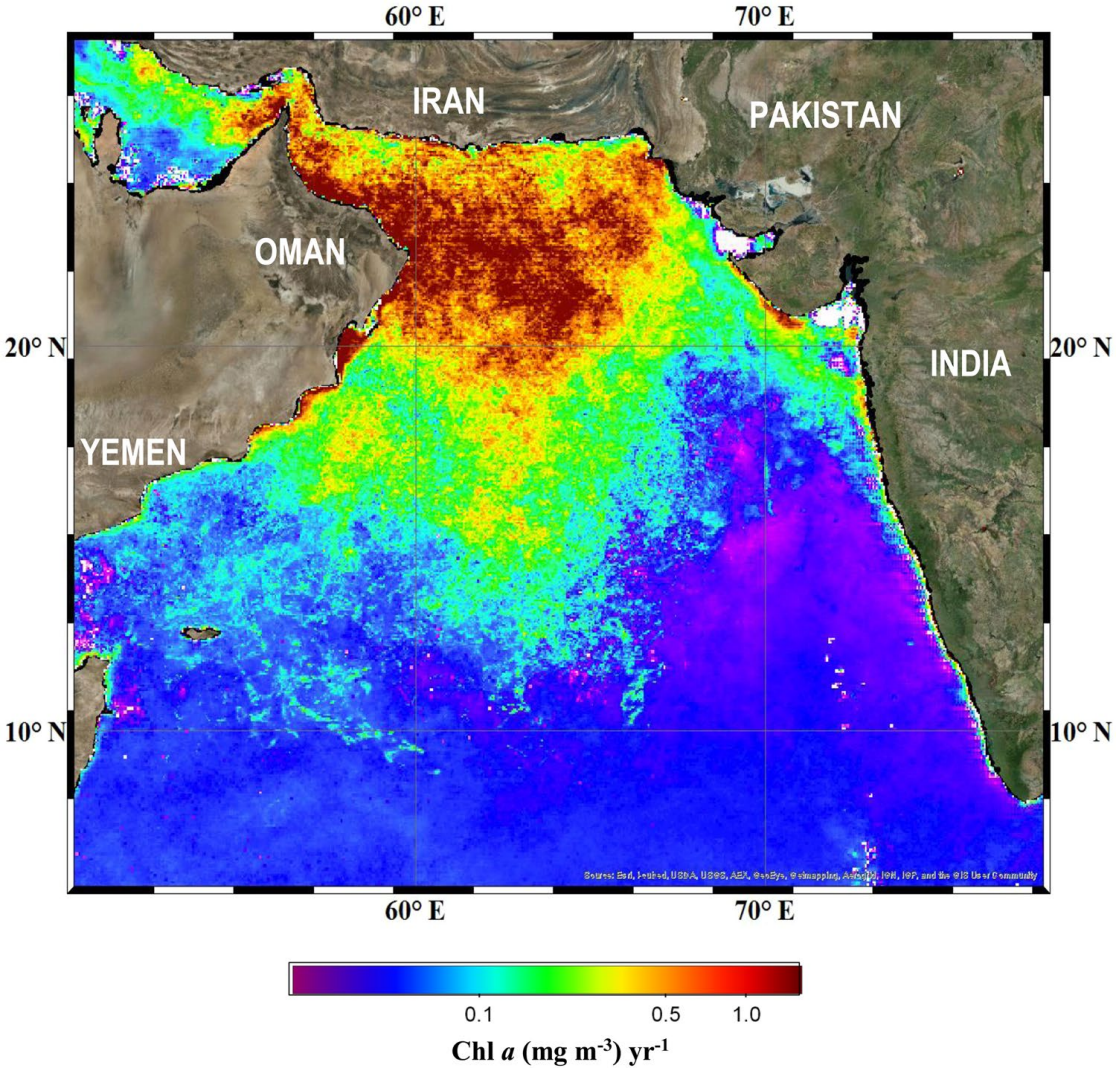
Spatial distributions of linear trends in winter-time Chl *a* in the AS for the period between 1996 and 2018*. (Additional details in methods section)*. Data processing was undertaken with GEE^59^.

Although a decline in winter biological productivity as forecast previously^20,22^ under the current scenario of weaker convective mixing and enhanced stratification seems like a realistic conjecture, trends in satellite-derived chlorophyll *a* (Chl *a*) concentrations present a different picture (Figs. 1c, 2). Since the early 2000s, winter-time Chl *a* concentrations averaged for the AS have been on the rise, increasing over 3-fold in recent years, particularly in the northwestern and central AS (Fig. 2), where winter Chl *a* concentrations now supersede those measured during the more productive summer monsoon season (Fig. S4). What is different, however, is that this increase in winter monsoon Chl *a* concentrations is not being fuelled by diatoms; the ubiquitous, trophically important, siliceous photosynthetic organisms, which dominated winter phytoplankton communities in the AS during the 1960s International Indian Ocean Expeditions (IIOE) and the mid-1990’s Joint Global Ocean Flux Studies (JGOFS)^4,28^. Rather, this is due to blooms of the mixotrophic green dinoflagellate *Noctiluca scintillans* Suriray (synonym *Noctiluca scintillans* Macartney)^29–34^. Since they were first detected in the early 2000s^29,31,32,35,36^ green *Noctiluca* blooms have become increasingly more pervasive over diatoms (Fig. S5) and more widespread, occurring every winter with predictable regularity^33,35–40^.

So how is a changing AS ecosystem, whose upper water column is becoming increasingly stratified and bordering nutrient limitation, benefiting *Noctiluca*? Here, we contend that changes in physico-chemical conditions in the euphotic water column caused by stratification, as well as *Noctiluca*’s mixotrophic mode of nutrition, act in tandem to give this organism a tremendous competitive advantage.

Firstly, as a mixotroph^31,35,41^, *Noctiluca* can meet its metabolic requirements via autotrophic CO_2_ fixation by thousands of free-swimming prasinophyte symbionts: *Protoeuglena noctilucae*^42^ within its central vacuole (symbiosome) (Fig. S6), and also via heterotrophic feeding on a wide range of external prey including phytoplankton, micro- and mesozooplankton and zooplankton eggs^36,41,43^. This mixotrophic mode of feeding gives green *Noctiluca* a trophic advantage over both autotrophic and heterotrophic plankton and makes it different from the globally ubiqutous red *Noctiluca* species, which are devoid of endosymbionts and exclusively heterotrophic^44^.

**Figure 3:**
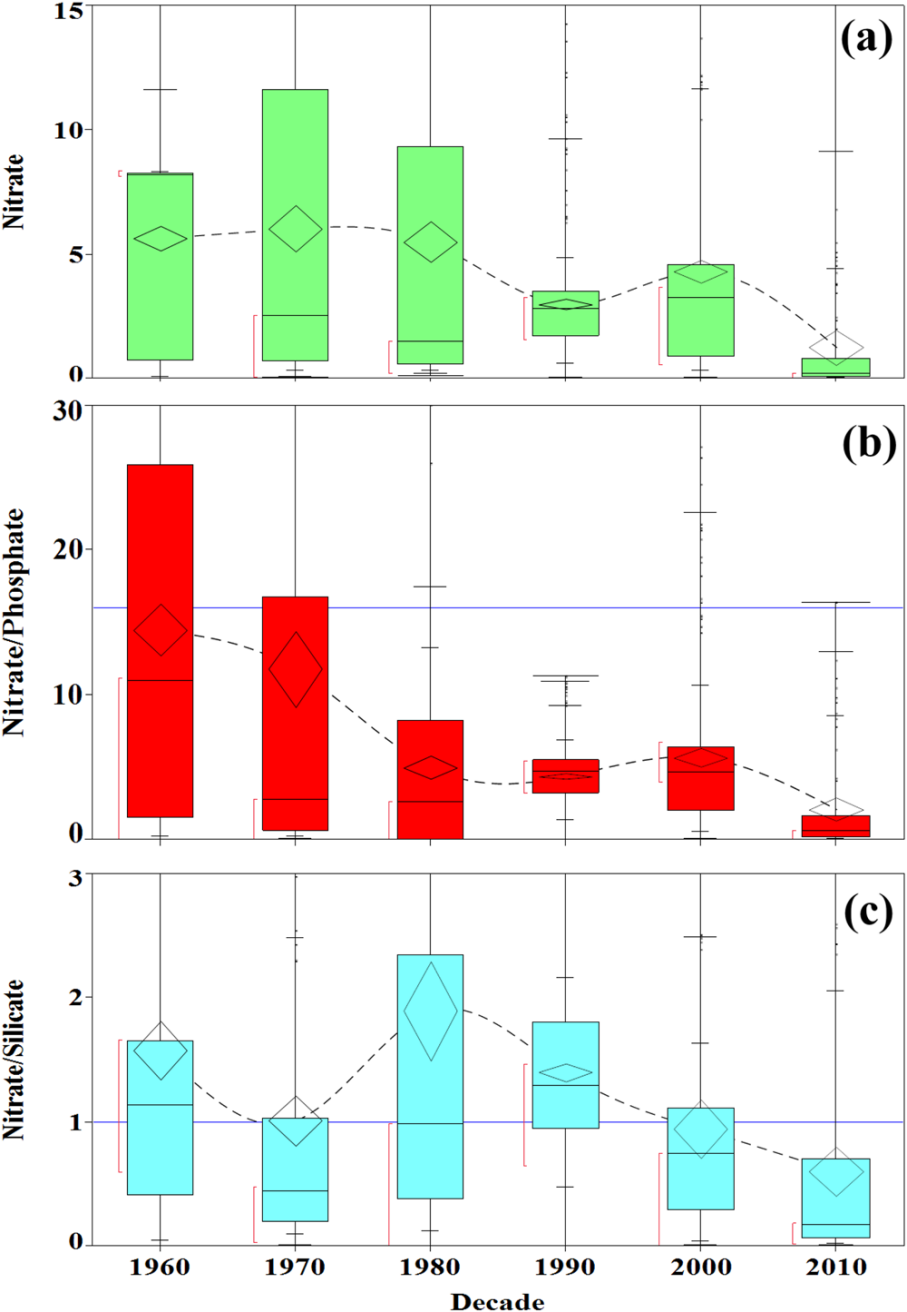
Decadal changes in (**a**) Nitrate, (**b**) Nitrate:Phosphate and (**c**) Nitrate:Silicate ratios in the AS. Blue horizontal lines in panels (**b**,**c**) represent typical seawater ratios (Redfield ratios) of 16:1 for NO_3_:PO_4_ and 1:1 NO_3_:SiO_3_, respectively. The ends of each box represent the 25th and 75th quantiles, respectively. The confidence diamonds contain the mean and the upper and lower 95% of the mean. The brackets to the left of each box identify the shortest half, which represent the densest 50% of the observations *(Additional details in methods section)*.

Secondly, our earlier work had ascribed the advent of *Noctiluca* blooms to the upshoaling of hypoxic waters in the euphotic zone^35^, caused by a possible expansion of the AS’s permanent oxygen minimum zone (OMZ)^45^. In this study^35^, we showed using shipboard experiments in which natural populations were exposed to suboxic seawater^36^ that endosymbionts in *Noctiluca* cells photosynthesized more efficiently under suboxic conditions. More recent studies^33,34,39^, have attributed the advent of *Noctiluca* blooms to acute “silicate stress”, a situation that would prevent diatom populations from attaining bloom proportions, particularly in the northwestern AS where they have been the predominant algal group in the past^28,29,32,46^. Our examination of winter-time nutrient concentrations coincident with *Noctiluca* blooms provides no evidence of silicate stress^31^, but instead raises the intriguing possibility that autotrophic phytoplankton in the contemporary AS ecosystem may in fact be experiencing acute “nitrate stress”. Analysis of nitrate concentration data, starting with the earliest measurements from the IIOE cruises of the 1960s up to more recent data collected during our *Noctiluca* bloom study cruises, show that the AS is experiencing a significant loss of nitrate inventories (Fig. 3a) within the upper euphotic column; a trend that in all likelihood, is being fostered by enhanced water column denitrification and ammonification on account of the AS’s expanding permanent OMZ^45^. Datasets of nutrient measurements from the early 1960s to date provide no evidence of any increases in inorganic phosphate in the AS, as would be expected from enhanced weathering of continental rocks in a warmer and more humid environment^47^. The decline in euphotic column nitrate concentrations and the profound departure in both NO_3_:PO_4_ and NO_3_:SiO_4_ ratios from traditional Redfield ratios (Fig. 3b,c) is unparalleled for any open ocean ecosystem. In this nutrient-poor scenario, we contend that as with other mixotrophs^48^, *Noctiluca’s* dual mode of obtaining nutrients^36^, confers upon it a significant competitive advantage over autotrophic phytoplankton especially when nutrients are limiting. When feeding on external prey, *Noctiluca* accumulates large amounts of nitrogen as ammonium (0.003–0.012 μM NH_4_^+^ cell^−1^) within its central cytoplasm^36^, alleviating its dependence on extraneous NO_3_. Feeding on external phytoplankton prey also reduces the standing stock of autotrophs competing for seawater nutrients, providing an additional advantage for *Noctiluca*. Additionally, in laboratory cultures illuminated on a diel cycle, we have consistently observed that *Noctiluca* grows best in medium enriched with ammonium. Laboratory-grown *Noctiluca* can also survive in the absence of nutrients and external prey for prolonged periods of time (~1 year), suggesting an internal, tight nutrient recycling mechanism that ensures its survival under conditions that would be considered hostile for most autotrophic phytoplankton.

Thirdly, *Noctiluca’s* ecological success and range expansion in the AS also appear to be tied to the lack of predatory pressure^35^. *Noctiluca’s* only known consumers are salps (Fig. S7) and jellyfish, which appear off the Omani coast in January, and in the central AS by March, long after large blooms of *Noctiluca* are established.

Finally, dinoflagellates, the functional group that *Noctiluca* belongs to, prefer less turbulent waters^49^ and typically reach their greatest abundance at the surface during relatively quiescent and nutrient-deplete periods^50^. This stands in contrast to diatoms, which with a few exceptions^47^ are generally restricted to more turbulent and nutrient-rich conditions^50,51^. In field studies, we have consistently noticed that a highly stratified and stable water column is favorable to the formation of *Noctiluca* blooms. This is evident along the coast of Oman, where although coastal upwelling shoals *Noctiluca*-favorable hypoxic waters into the upper euphotic zone as early as Aug. (Fig. S8), accumulation of *Noctiluca* to bloom proportions takes place only in sheltered coastal, embayments, when the summer monsoon winds ease^29^ and the water column becomes more stable than offshore waters. Offshore in the AS, prior to their appearance as surface blooms in Dec-Jan, *Noctiluca* are found at depth^31^, often close to the oxycline and where photosynthetically available radiation (PAR) is <100 μmol (photons) m^−2^ s^−1^^31^. Surface *Noctiluca* blooms appear by early Jan.^31^, when the water column begins to stratify, under high light conditions that are typical at that time of the year. Then in the presence of extraneous prey, they transition to a greater dependence on heterotrophy to attain the high growth rates of ~1.2 cells day^−1^^ 35,36^ necessary for bloom formation.

Besides the build-up of ammonium^31,36^ mentioned earlier, *Noctiluca* can accumulate significant amounts of lipids in its central cytoplasm when feeding on extraneous prey^31^, which makes individual cells increasingly buoyant and thus easily prone to dispersal by surface currents, filaments and streamers associated with mesoscale eddies^52^, allowing it to become more widespread (Fig. 2).

In summary, we contend that while *Noctiluca* outbreaks are triggered each summer by the intrusion of hypoxic waters into the upper layers of the euphotic column^35^, conditions under which *Noctiluca*’s endosymbionts have shown to have higher rates of photosynthesis than free-living autotrophs. Stratification and a stable water column aid in enhancing their growth to large bloom proportions. In the latter situation, *Noctiluca*’s unique mixotrophic mode of obtaining nutrition offers it considerable advantages over autotrophs, which are severely nutrient limited by a weaker convective mixing due to the loss of snow cover in the HTP, and changes in nutrient stoichiometries caused by the expansion of the OMZ. Under these conditions, *Noctiluca*’s unique symbiotic system provides it with a tight nutrient recycling mechanism; wherein nitrogenous nutrients, accumulated during digestion of ingested prey, help alleviate nutrient limitation in the external environment.

A recent study^53^ has drawn attention to an increase in anthropogenic organic carbon exiting the northern Arabian (Persian) Gulf that may be contributing significantly to the expansion of the OMZ in the northwestern AS, where *Noctiluca* blooms have been particularly more intense (Fig. 2).

Recent observations of *Noctiluca* blooms, in association with the shallowing of hypoxic waters along the coast of Oman during the summer upwelling season^30^, have raised the specter that this organism may also be expanding its temporal range to include the highly productive summer period when diatom blooms support Oman’s large coastal artisanal fisheries. In under-developed countries like Somalia and Yemen, which are currently being challenged by unrest, poverty and deprivation^54,55^, and where fisheries are the primary source of protein and income, any further loss of fishery resources, due to the spread of hypoxia (Fig. S8), has the potential to further exacerbate socio-economic turmoil in the region, including piracy on international shipping^56^.

The inability of large zooplankton, except salps and jellyfish to feed on *Noctiluca*, is indicative of the capacity of *Noctiluca* blooms to short-circuit the trophic food chain. Thus, their annual reoccurrence and growing dominance in winter each year will require a revision of our fundamental understanding of the AS food web garnered from the AS-JGOFS era^6^, when autotrophs dominated during productive seasons and alternated with diazotrophs during the transition to nutrient limiting conditions of the inter-monsoon seasons^28^. The emergence of a mixotroph as a dominant organism will also necessitate revisions to the traditional manner in which AS carbon cycling and biogeochemical rate processes are modeled and studied. Current models compartmentalize organisms at the base of the food chain into autotrophic phytoplankton and heterotrophic zooplankton, but mixotrophy blurs the strict boundary between producers and consumers^48,57,58^. Recent model simulations^58^ that include mixotrophs have indicated that mixotrophy greatly enhances the transfer of biomass to larger size classes further up the food chain, which is consistent with our observations of large amounts of salps, jellyfish, and squid in the AS, particularly along the coast of Oman in association with *Noctiluca* blooms.

## Methods

Mixed layer depth anomalies were calculated using area averaged (60°E–70°E, 14°N–25°N) mixed layer data for the months of Jan-Feb-Mar data from Global Ocean Data Assimilation System (GODAS) model of NOAA, Oceanic and Atmospheric Research, Earth System Research Laboratory, Physical Sciences Division. GODAS is a real-time ocean analysis and a reanalysis system, forced by the momentum flux, heat flux and fresh water flux. It assimilates temperature and synthetic salinity profiles from NCEP Atmospheric Reanalysis 2. Mixed Layer Depth is a product of the K-Profile Parameterization (KPP) Mixed Layer Scheme and is the depth where the buoyancy difference with respect to the surface is equal to 0.03 cm s^−2^. The data are available at 1/3^o^ × 1/3^o^. The period used for calculating the anomalies is from 1980 to 2018.

Air temperature anomalies over the AS were calculated using the area averaged (50°E–77.5°E, 8°N–25.6°N) air temperature data from the NOAA, National Centers for Environmental Prediction, Department of Energy (NCEP/DOE) Reanalysis-2 project. The data used are for the months of Jan-Feb-Mar, and available from the NOAA, National Centers for Environmental Information. The data period used for calculating the anomalies is from 1949 to 2018, and the plot is from 1970 to 2018.

Wind speed anomalies over the AS were calculated using the area averaged (50°E–77.5°E, 8°N–25.6°N) wind speed data for the months of Jan-Feb-Mar, obtained from NOAA, Climate Data Record, Ocean Near-Surface Atmospheric Properties, Version 2. The data period used for calculating the anomalies is from 1988 to 2017.

Relative humidity (RH) anomalies over the AS were calculated using area averaged (50°E–78°E, 8°N–25.6°N) RH data for the months of Dec-Jan-Feb, obtained from NASA MERRA 3D IAU State, Meteorology Monthly Mean V5.2.0. The data period used for calculating the anomalies is from 1980 to 2016.

Net Heat Flux anomalies over the AS were calculated using the area averaged (50°E–77.5°E, 8°N–25.6°N) net heat flux data for the months of Jan-Feb-Mar, from NOAA, NCEP-NCAR Reanalysis 2. The data period used for calculating the anomalies is from 1960 to 2017.

Eurasian snow cover extent anomalies were calculated using area averaged (60E°–80°E, 20°N–40°N) snow cover extent data obtained from NASA, National Snow and Ice Data Centre (NSIDC), Northern Hemisphere EASE-Grid 2.0 Weekly Snow Cover and Sea Ice Extent, Version 4. The data period used for calculating the anomalies is from 1967 to 2018.

Trend lines and *p* values shown in Figs. 1a–c, S1a–d are based on ordinary least square fits to the time series data.

Area averaged (80°E–120°E, 20°N–40°N) air temperature anomalies over Eurasia for the period were obtained using air temperature data from 1948 to 2016 obtained from the Climate Research Unit Temperature (CRUT) Version 4.01, University of East Anglia, UK.

Area averaged (50° to 77.5°E, 8° to 25.6°N) Chl *a* concentrations as Level 3 products for winter (Jan-Feb-Mar) were obtained from NASA CZCS (Oct.1978 to Jun. 1986, Version 2014.0), Japan National Space Development Agency OCTS (Nov 1996 to Jun. 1997, Version 2014.0), NASA SeaWiFS (Sept 1997 to Dec. 2010, Version 2018.0) and NASA MODIS-Aqua (Jul. 2002 to present, Version 2018.0). For the period between Jul. 2002 and Dec. 2010, when SeaWiFS and MODIS-Aqua overlapped, the data presented are based on the means of the two data sets. All datasets have been processed by the Ocean Biology Processing group at NASA Goddard Space Flight Centre (GSFC). These daily products have been corrected for atmospheric light scattering and for sun angles differing from the nadir. In addition, the influence of clouds has been substantially reduced. To account for sensor degradation over time, the instrument is calibrated using internal lamps, solar diffuser observations, and lunar images, as well as vicarious methods.

Seawater nutrient data for estimating decadal changes in winter time (Dec-Jan-Feb-Mar) NO_3_:PO_4_ and NO_3_:SiO_3_ ratios (55°E–76°E, 8°N–24°N) in the upper 80 m of the AS were obtained from NOAA, National Centers for Environmental Information, USA, Indian National Oceanographic Data Centre, India, National Science Foundation, Biological and Chemical Oceanography Data Management Office, USA, British Oceanographic Data Centre, UK and oceanographic cruises of the Ministry of Earth Sciences, Govt. of India. The data period ranged between 1965 and 2011.

## Supplementary information

Supplementary Material.
