# Supplementary material for: Ecosystem state change in the Arabian Sea fuelled by the recent loss of snow over the Himalayan-Tibetan Plateau region
Source: Sci Rep. 2020 May 4;10:7422. doi: 10.1038/s41598-020-64360-2 (PMC7198515; doi:10.1038/s41598-020-64360-2)
Supplement: Supplementary file 1 — Supplementary Material. [file 41598_2020_64360_MOESM1_ESM.pdf]

SUPPLEMENTARY MATERIAL

Ecosystem state change in the Arabian Sea fuelled by the recent loss of snow over the Himalayan-Tibetan Plateau region

Joaquim I. Goes, Hongzhen Tian, Helga do Rosario Gomes, O. Roger Anderson, Khalid Al-Hashimi,

Sergio deRada, Hao Luo, Lubna Al-Kharusi[5], Adnan Al-Azri, Douglas G. Martinson

All correspondence should be addressed to J.I.G at E-mail: *jig@ldeo.columbia.edu*

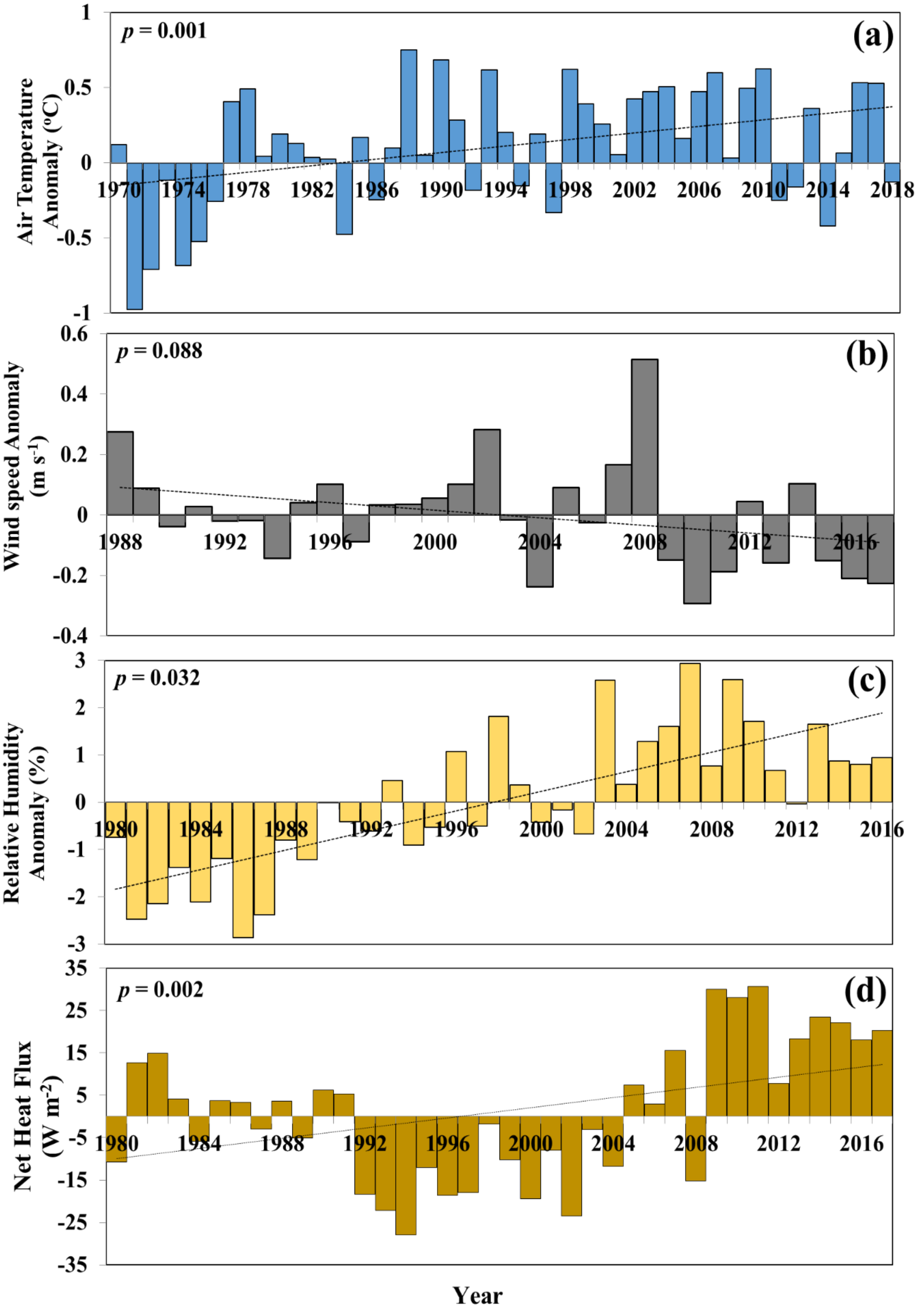

Fig. S1a-d Time series of winter-time, AS area averaged anomalies (departures from means) of (a) air temperature, (b) wind speed, (c) relative humidity and (d) net heat flux. Trends (dashed lines) were obtained using linear least square regression fits to the data. Also shown are *p* values associated with individual trends

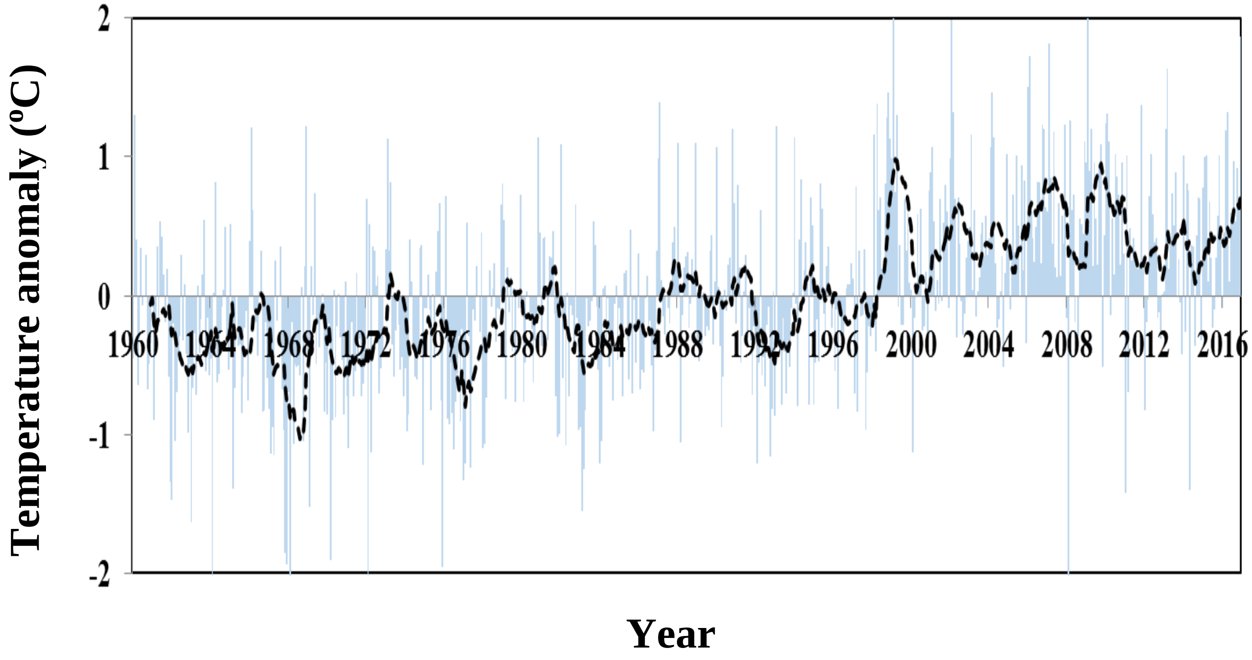

Fig. S2 Area averaged air temperature anomalies over Eurasia for the period between 1960 and 2016. *(Additional details in methods section).*

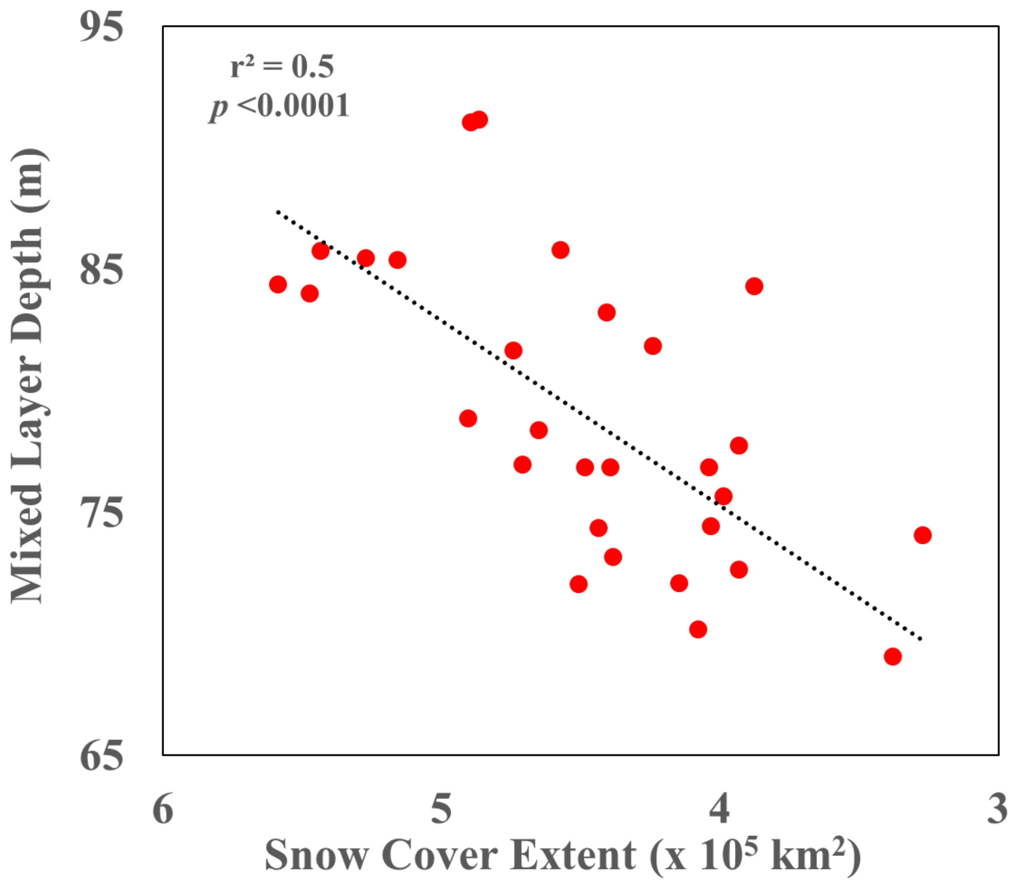

Fig. S3 Scatter plot of snow cover extent versus mixed layer depth *(Additional details in methods section)*

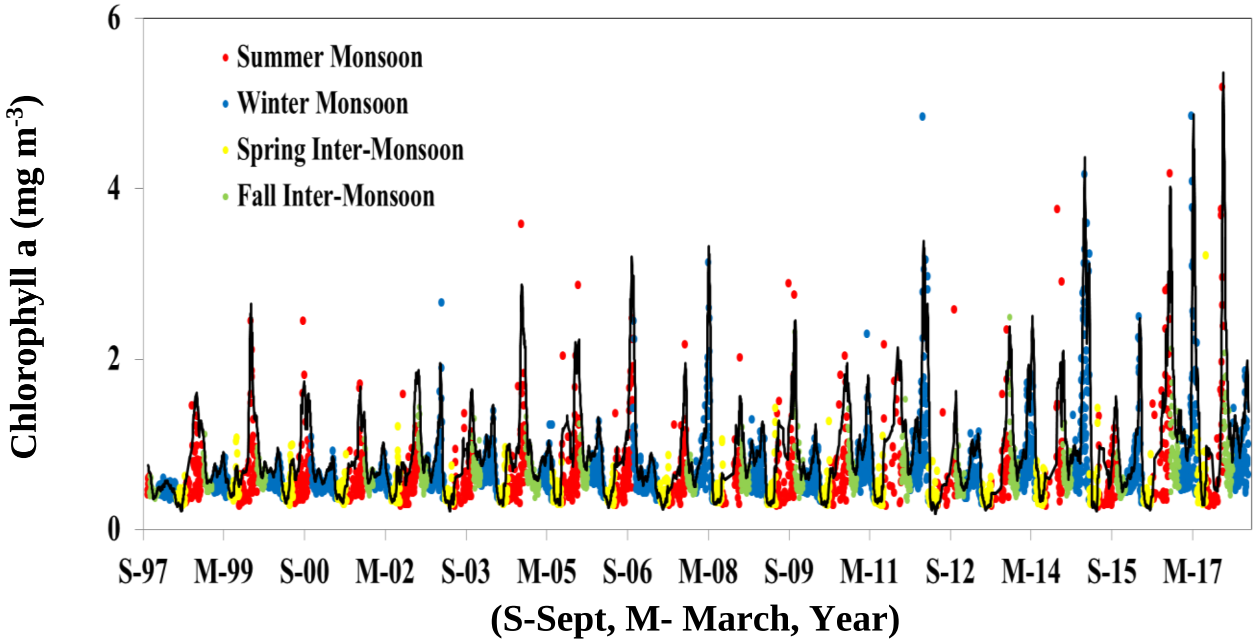

Fig. S4 Interannual changes in satellite-derived summer and winter monsoon peaks of Chl *a* for the period from Sep. 1997 to Sep. 2017. *(Additional details in methods section).*

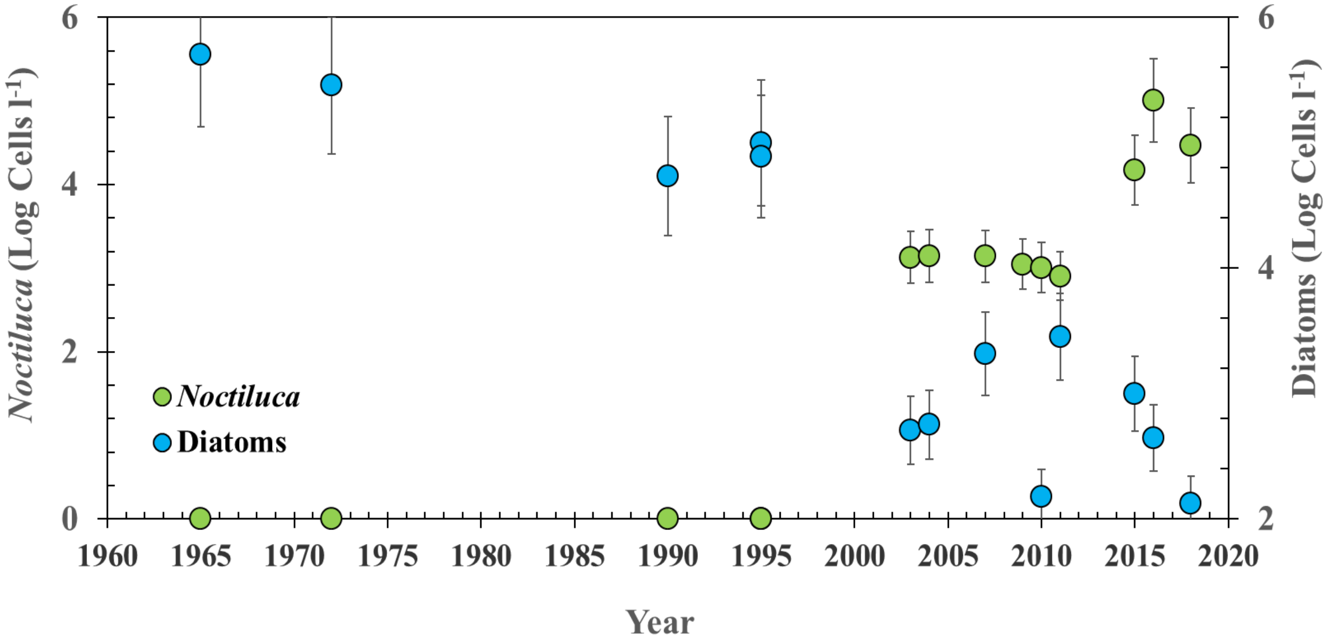

Fig. S5 Changes in total counts of *N. scintillans* and diatoms from cruises in the Arabian Sea north of 19°N. Data from 1960 to 2011 were compiled by us and presented previously in Gomes et al. (2014) [35]. Data after 2011 are from blooms encountered by Sarma et al (2019) [39] in 2015, Baliarsingh et al. (2018) [38] in 2016 and Xiang et al. (2019) [40] in 2018. All values are presented in Log Cells l^{-1}.

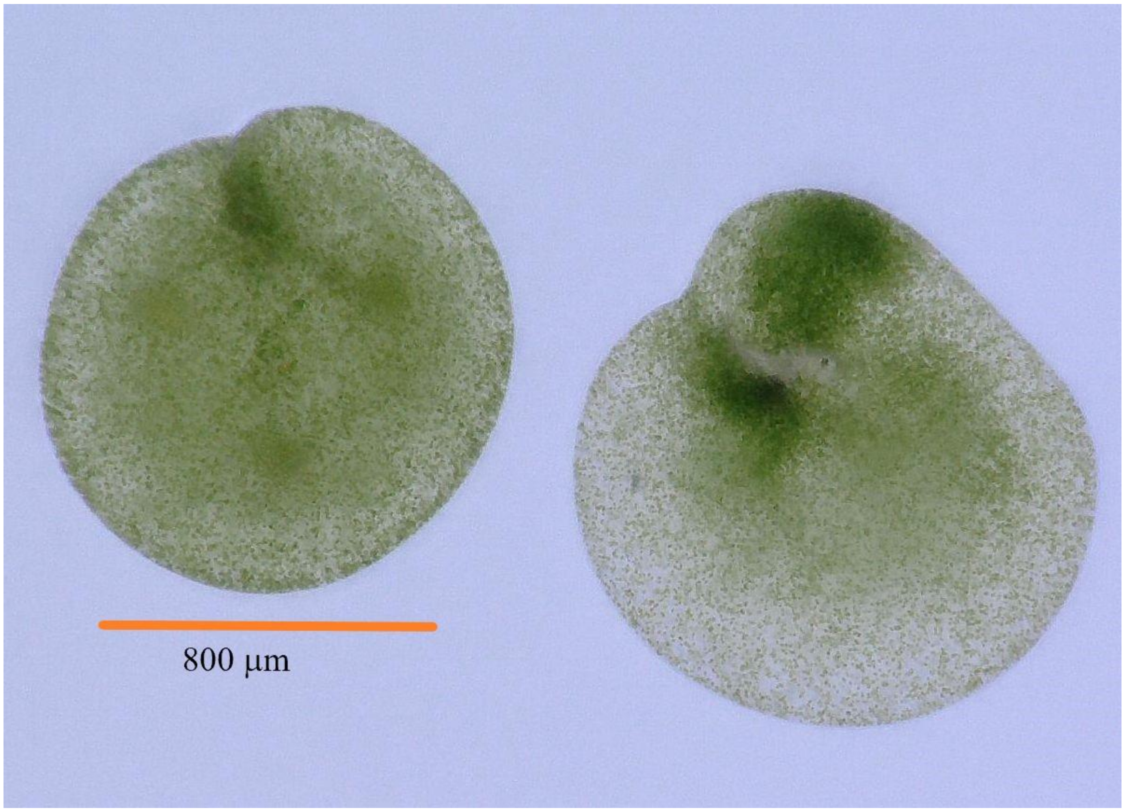

Fig. S6 Microscopic image of *Noctiluca* cells showing thousands of cells of *Protoeuglena noctilucae* within central cytoplasm (symbiosome).

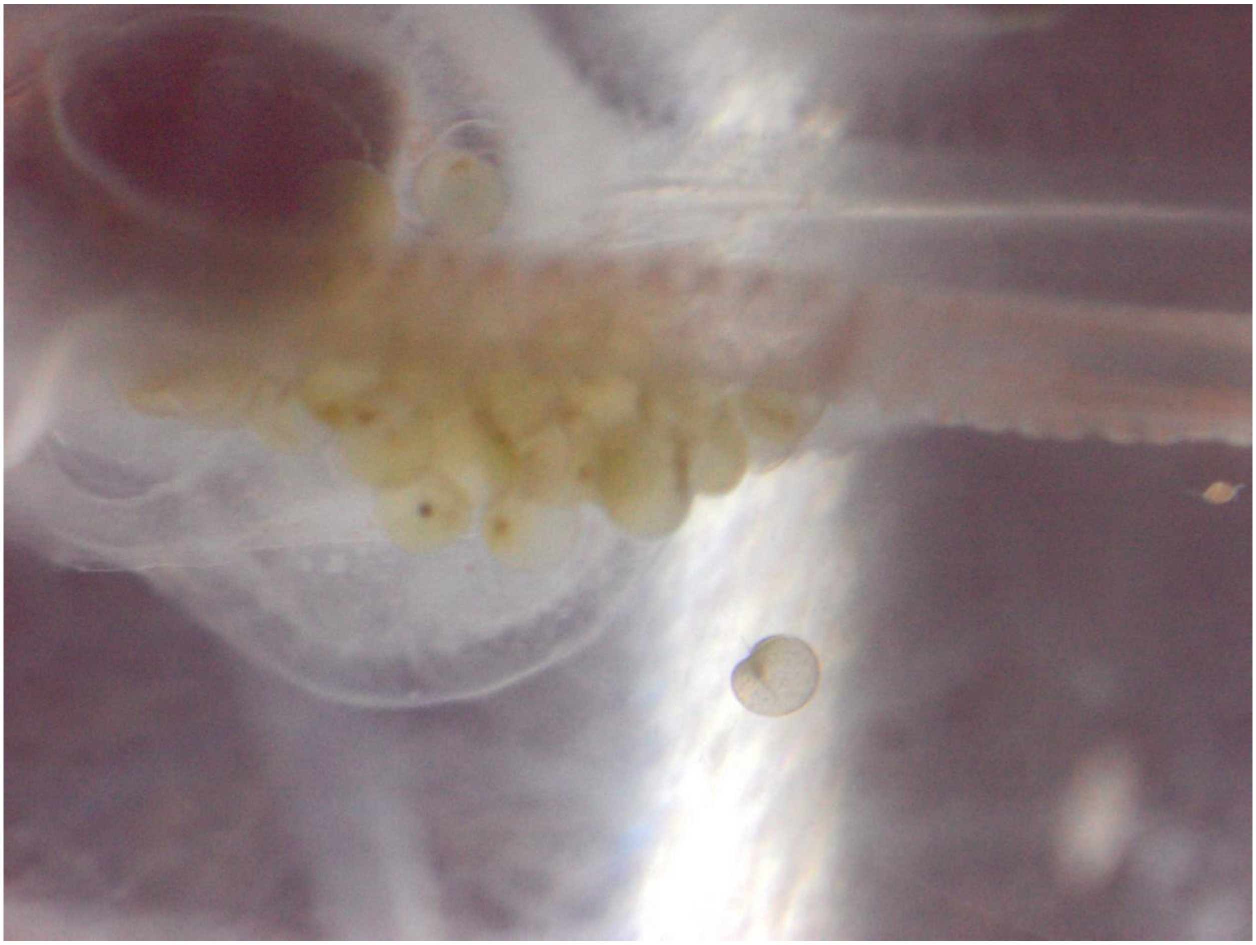

Fig. S7 Stereomicroscope image of a salp showing the presence of *Noctiluca* cells within its digestive system.

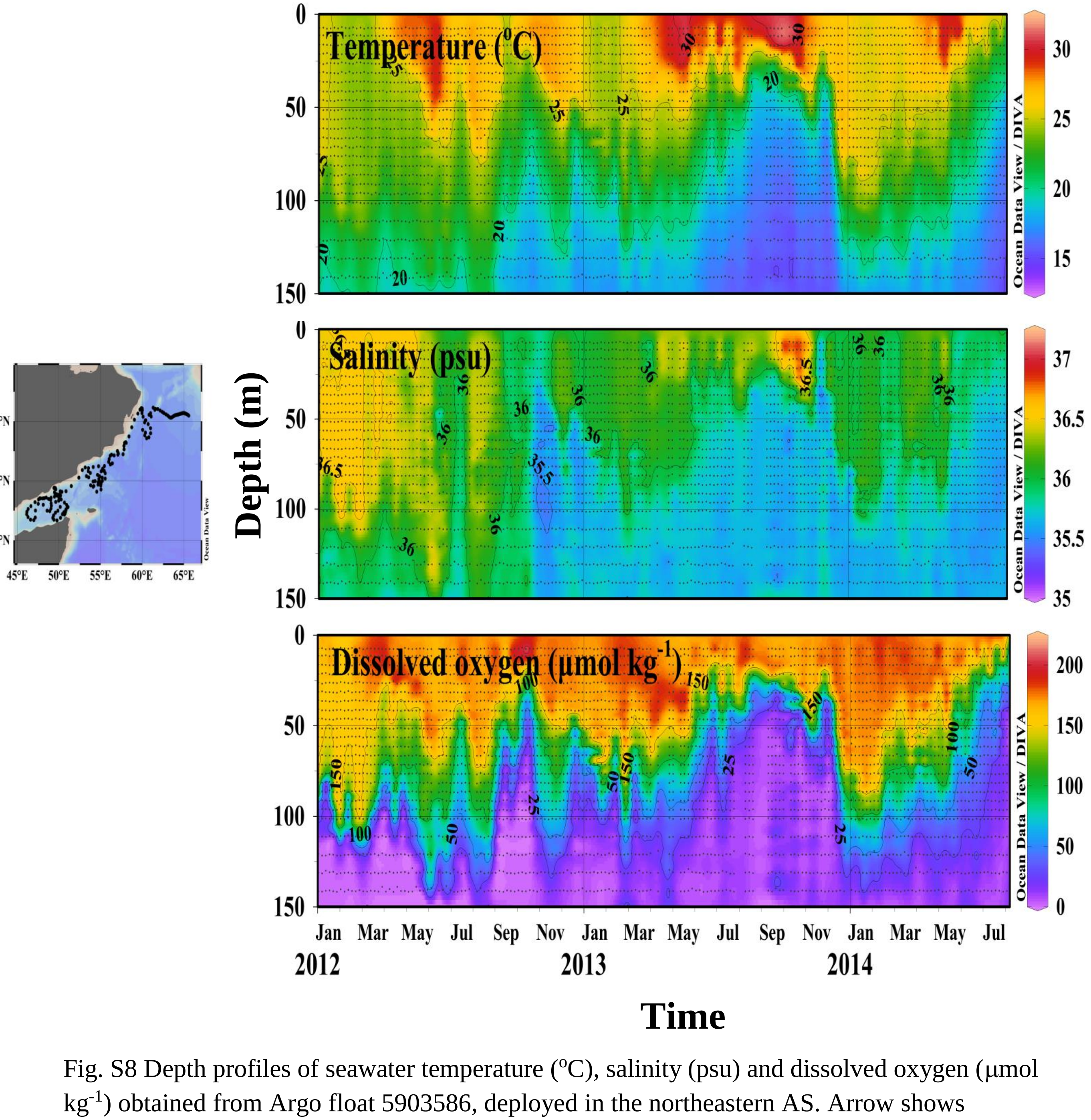

Fig. S8 Depth profiles of seawater temperature (ºC), salinity (psu) and dissolved oxygen (µmol kg^{-1}) obtained from Argo float 5903586, deployed in the northeastern AS. Arrow shows trajectory of the float. Data were plotted using Ocean Data View[1] and dots represent depth at which measurement was recorded *(Additional details in methods section).*

1. Schlitzer, R., Ocean Data View, odv.awi.de, 2018..
